# Case Report: Unraveling complex genomic alterations in a case of chronic lymphocytic leukemia using optical genome mapping

**DOI:** 10.3389/fonc.2025.1639849

**Published:** 2025-09-03

**Authors:** Leila Youssefian, Trilochan Sahoo, Jia-Chi Wang

**Affiliations:** 1 Cytogenetics Laboratory, City of Hope National Medical Center, Irwindale, CA, United States; 2 Bionano Laboratories, San Diego, CA, United States

**Keywords:** fluorescence *in situ* hybridization (FISH), chronic lymphocytic leukemia (CLL), optical genome mapping (OGM), chromothripsis and chomoplexy, complex karyotype (CK), structural variants (SV), genome instability, cancer cytogenetics

## Abstract

Chronic lymphocytic leukemia (CLL) is one of the most prevalent adult leukemias, derived from mature B-cells and exhibiting a highly heterogeneous disease course. Standard cytogenetic analysis of CLL includes FISH and karyotyping. However, conventional chromosome analysis of cancer specimens is often constrained by low chromosomal resolution, and FISH analysis is limited by the number of probes that can be applied. This study highlights the application of optical genome mapping (OGM), a high-resolution cytogenomic tool that visualizes ultra-long, sequence-labeled DNA molecules, to uncover the structural complexity of the cancer genome and assess the clinical relevance of chromothripsis in CLL. Comprehensive cytogenetic analysis was conducted on a 43-year-old male diagnosed with chronic lymphocytic leukemia. Karyotyping revealed a complex rearrangement: 46,XY,der(3)t(3;13)(p2?3;q14.3),der(4)t(?3;4)(p23;p16),add(11)(p13),del(13)(q14)[12]/46,sl,del(11)(q2?2.2q23.3)[6]. FISH analysis further identified the loss of ATM and a partial deletion of the D13S319 locus. OGM analysis performed on bone marrow revealed a complex genotype including chromothripsis of chromosome 13, and structural rearrangements involving chromosomes 3, 4, and 11. Additionally, multiple intrachromosomal translocations and interstitial microdeletions of chromosome 13 were identified. The resolution of these aberrations has been significantly enhanced with examples including: ogm[GRCh38] t(3;13)(p26.3;q33.1)(2,706,645~2,721,113;103,142,901~103,154,241][VAF0.45], ogm[GRCh38] t(4;13)(p15.31;q32.1)(20,869,721~20,907,265;96,617,837~96,630,317)[VAF0.42],. In conclusion, OGM revealed the intricate structural alterations of the cancer genome. The high resolution provided by OGM could facilitate the discovery of oncogenic mechanisms, novel fusion genes, prognostic markers, and potential therapeutic targets. OGM serves as a powerful tool for revisiting CLL disease classification by offering deeper insights into complex genomic rearrangements.

## Introduction

Cancer development is driven by stepwise genomic alterations, including complex chromosomal rearrangements known as chromoanagenesis, that promote clonal evolution and malignant transformation. Chromoanagenesis, used as an umbrella term, refers to catastrophic one-step genomic rearrangements, including chromothripsis, chromoplexy, and chromoanasynthesis (1, 2). These rearrangements are generated by different mechanisms and have been associated with various cancers and/or constitutional disorders (2–4). Chromothripsis and chromoplexy result from faulty DNA repair via Non-Homologous End Joining (NHEJ), while chromoanasynthesis arises from replication-based mechanisms like fork stalling and template switching (FoSTeS) or microhomology-mediated break-induced replication (MMBIR) (5–7). Chromothripsis is a catastrophic event causing extensive chromosome fragmentation and rearrangement, marked by at least seven copy number changes on one or a few chromosomes (5, 8, 9). Approximately a decade ago, chromothripsis was first identified in the genome of a CLL patient through massively parallel, paired-end sequencing. This analysis revealed disruptions in the long arm of chromosome 4, as well as focal genomic regions on chromosomes 1, 12, and 15 (5), and subsequently in different tumors with a prevalence of 30-50% across all cancer subtypes (3). Chromothripsis challenges traditional tumorigenesis models by causing abrupt chromosome shattering and reassembly, leading to rapid genomic imbalance and aggressive tumor behavior. Chromothripsis in CLL patients with complex karyotypes has been reported in several studies (5, 9–12). However, the combined occurrence of chromothripsis and chromoplexy is rare in CLL and has been reported in only a few cases (12, 13).

Advances in technology have ushered the ‘omics’ era into both research and clinical practice, with comprehensive DNA sequencing routinely used for diagnosing and identifying actionable findings in constitutional and cancer genomics. Yet, a dichotomy remains in clinical laboratories: while much focus has been placed on detecting single nucleotide variants (SNVs), structural variant (SV) analysis still relies on traditional cytogenetic methods—chromosome banding analysis (CBA), fluorescence *in situ* hybridization (FISH), and chromosomal microarrays (CMA)—each with well-known diagnostic and resolution limitations (5–10 Mb for CBA, 70 kb–1 Mb for FISH, and 5–200 kb for CMA) (14). This underscores the need for more comprehensive tools to reliably detect clinically significant SVs often missed by conventional methods.

Optical Genome Mapping (OGM) is an emerging high-resolution cytogenomic technology designed to detect SVs and unravel complex chromosomal rearrangements such as those caused by chromoanagenesis. By imaging ultra-long DNA molecules labeled at specific sequence motifs, OGM provides a genome-wide view of large-scale structural changes—including translocations, inversions, insertions, deletions, duplications, and complex rearrangements like chromothripsis and chromoplexy. It offers high sensitivity, faster turnaround time, no need for cell culture, and superior resolution (~500 bp) for detecting cryptic and complex SVs. However, OGM cannot detect SNVs, small indels, or methylation changes, and it may face challenges in highly repetitive regions. Outputs include detailed SV maps, breakpoint visualization, and automated variant classification to support both clinical and research applications (15, 16).

We report a case of 43-year-old male diagnosed with CLL, where integrative studies using conventional and molecular cytogenomics, including optical genome mapping (OGM) technology, revealed chromothripsis on chromosome 13 along with chromoplexy involving chromosomes 3, 4, 11 and 13. This event has been previously described in only a few CLL cases (12, 13). The OGM technique has proven to be an excellent tool, that is based on imaging of long DNA molecules labeled at specific sites to identify multiple cytogenetic abnormalities in a single test. OGM is a robust technology that can be implemented in the routine management of CLL patients, offering more precise disease classification and risk stratification. However, further studies are required to define standardized criteria for genomic complexity.

## Materials and methods

### Conventional chromosome analysis

A total of 200 µl buffy coat from the bone marrow specimens was added into each cell culture, with the stimulation of DSP30 and/or IL-2 for 72 hours or in BM-Condimed media for 48 hours before harvest. The chromosomes were banded using standard Giemsa banding procedure. Analysis and karyotyping are carried out by two certified technologists using the ASI automated metaphase scanner and software. Both technologists analyze the first 10 consecutive metaphases after which they are reviewed by the pre-reviewer and reported by the director.

### Fluorescence *in situ* hybridization

FISH analysis was performed with commercially available probes for CLL. The slide with added probe mixture was co-denatured at 73°C for 3 min and hybridized overnight at 37°C using either Vysis HYBrite or Abbott ThermoBrite hybridization system. The slide was washed next day in 0.4xSSC/0.3% IGEPAL CA-630, pH 7 at 73°C for 2 min, and in 2xSSC/0.1% IGEPAL CA-630, pH 7 at room temperature for 30 sec, counterstained with DAPI, and visualized under fluorescent microscope.

### Optical genome mapping

OGM analysis was carried out by Bionano Genomics laboratory (San Diego, CA, USA). The steps were followed in accordance with the standard procedures (Bionano Prep^®^ SP BMA DNA Isolation Protocol v2, 2021, Bionano Genomics). In brief, the bone marrow aspirate, collected from the same bone marrow specimen initially used for karyotyping in October 2020 before the initiation of clinical treatment and pre-treated with DNA stabilizer, was filtered and centrifuged. The pelleted WBC was lysed and treated with Proteinase K and RNAse in the Lysis and Binding Buffer (LBB) and incubated in the phenylmethylsulphonyl fluoride solution (PMSF). Ultra-high molecular weight (UHMW) genomic DNA was bound to the nanobind thermoplastic paramagnetic disk to prevent fragmentation, washed by a series of washing buffers, eluted, and homogenized by pipette mixing with a 200 μl standard pipette tip. The UHMW DNA was then quantified using the Qubit dsDNA Broad Range Assay Kit (Thermo Fisher Scientific). A total of 750 ng UHMW genomic DNA with concentration of 200–300 ng/μL underwent sequence-specific fluorescent labeling using a Direct Label Enzyme (DLE-1). The DLE-1 enzyme attaches the DL-Green fluorophore via covalent modification at a specific-sequence motif. The labeled DNA was digested by Proteinase K, cleaned up, homogenized, stained and quantitated. The expected metrics of the UHMW DNA are N50 (> 150 kbp) > 230 kbp, Labels/100 kbp: 14-17, Map Rate > 70%, Positive Label Variance < 10%, Negative Label Variance < 15%. The equilibrated and labeled sample was loaded on Saphyr Chip following the standard procedures of Saphyr^®^ System User Guide (Bionano Genomics). The data was analyzed, generated, and loaded in Bionano Access^®^, re-exported by Bionano VIA^®^ software, which enables users to view Saphyr^®^ run results in real time and perform a variety of bioinformatics analyses.

### Patient’s clinical and other laboratory information

A 43-year-old Caucasian male of Russian, Ashkenazi and Persian descent was diagnosed with CLL after an evaluation for elevated lymphocyte count in May 2019. At that time, the CBC showed a WBC of 37,500 cells per microliter with 18% neutrophils, 74% lymphocytes, and an absolute lymphocyte count of 29,600. A review of his lab results revealed a consistent increase in his WBC count over the years: 6.8 K/µL in 2014, 8.4 K/µL in 2016, 13.0 K/µL in 2017, 37.5 K/µL in 2019 and 23.6 K/µL in 2024. The flow cytometry revealed that 70% of his blood mononuclear cells co-expressed CD19, CD20 dim, CD5, CD23, CD38, and HLA-DR, and surface immunoglobulin kappa, but negative for FMC7, CD103 and CD10. He subsequently enrolled on a clinical trial with obinutuzumab and ibrutinib started in October 2020 and discontinued in August 2021 after he was found to be MRD negative. In October 2024, the patient remained asymptomatic with nonsignificant clinical findings. Physical examination was unremarkable, with no evidence of hepatosplenomegaly or lymphadenopathy. At the follow-up visit in November 2024, the patient developed thrombocytopenia and additional clinical signs, leading to classification as Rai stage 4. In response, treatment with ibrutinib and obinutuzumab was reinitiated. Molecular studies, including *IGHV* mutation analysis and a targeted NGS panel for hematologic malignancy genes, consistently revealed an unmutated *IGHV* rearrangement and wild-type *TP53* at both initial diagnosis and follow-up evaluations.

## Results

### G-banded chromosome and FISH analysis

**Figure 1:**
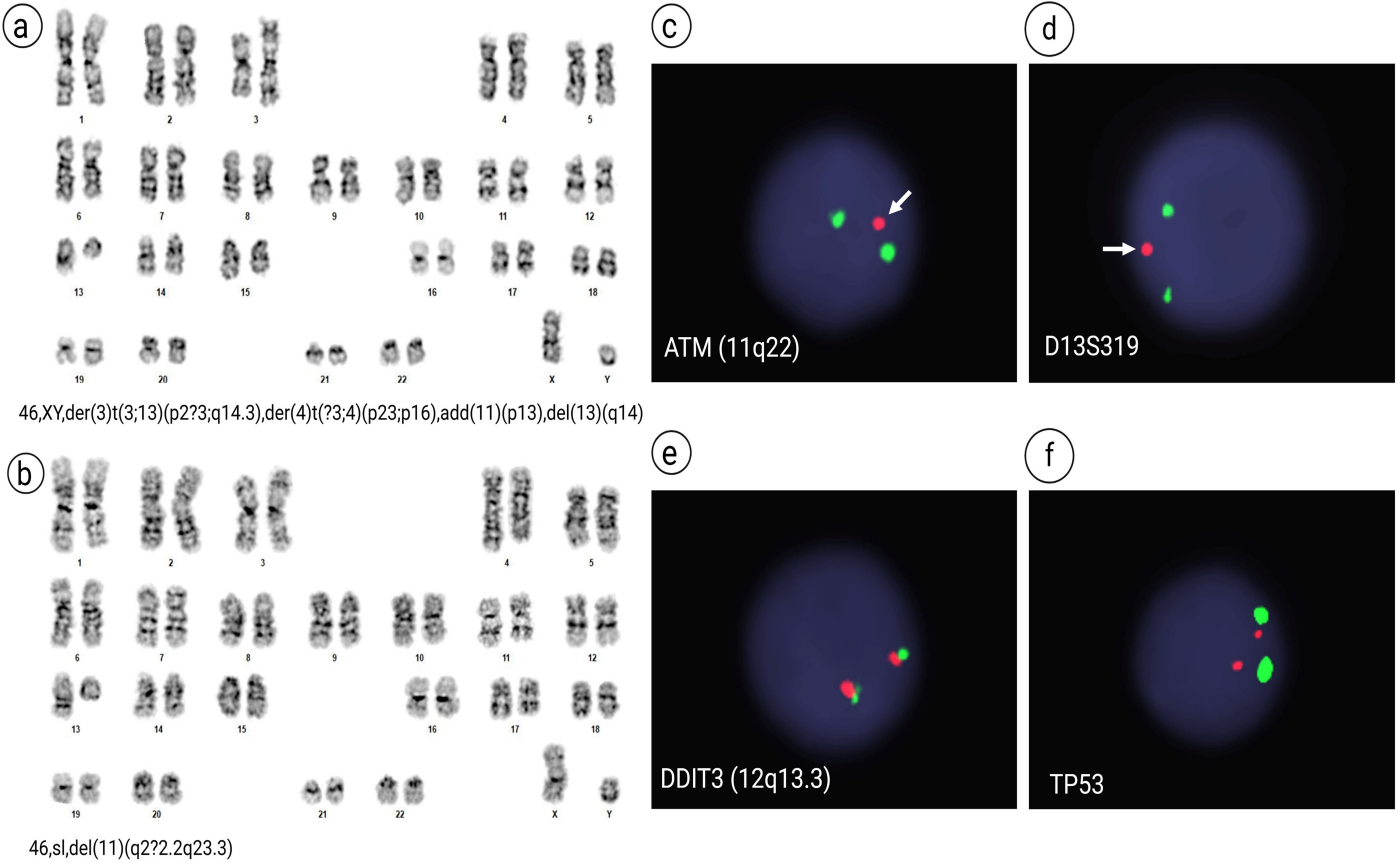
G-banded Chromosome and FISH analyses. Karyotype analysis revealed two related abnormal clones in 18 out of 20 cells examined. **(a)** The stemline, 46,XY,der(3)t(3;13)(p2?3;q14.3),der(4)t(?3;4)(p23;p16),add(11)(p13),del(13)(q14)[12], and **(b)** six sideline cells, 46,sl,del(11)(q2?2.2q23.3)[6]. FISH analysis revealed: **(c)** loss of the *ATM* signal (red) at 11q22.3, with only one *ATM* signal (arrow) observed in 93% of interphase nuclei (186/200), indicated by the absence of the red signal paired with the green centromeric control probe (D11Z1; CEP11); **(d)** monoallelic loss of D13S319 at 13q14.3 (arrow) in 65% of nuclei (130/200), with a red signal for D13S319 and green for the control probe *LAMP1*; **(e)** a negative signal pattern for trisomy 12 using the *DDIT3* probe set (red signal for the 3′ centromeric region and green for the 5′ telomeric region at 12q13.3); and **(f)** intact signals for *TP53*, with red at 17p13.1 and green at the centromere (D17Z1; CEP17), in all 200 nuclei examined.

Conventional chromosome analysis of 20 cells performed in October 2020 revealed that 18 mitotic cells were clonally abnormal, comprising two related abnormal populations, while the remaining two cells appeared cytogenetically normal. The stemline, 46,XY,der(3)t(3;13)(p2?3;q14.3), der(4)t(?3;4)(p23;p16),add(11)(p13),del(13)(q14)[12], composed of twelve cells, was characterized by a derivative chromosome 3 resulting from an unbalanced translocation between the short arm of chromosome 3 and the long arm of chromosome 13, a derivative chromosome 4 suspected to result from an unbalanced translocation between distal short arm of chromosome 4 with the distal short arm of chromosome 3, additional material of unknown origin on the short arm of chromosome 11, and a derivative chromosome 13 arising from the unbalanced translocation with chromosome 3 (**Figure 1a**). Six sideline cells, 46,sl,del(11)(q2?2.2q23.3)[6], contained an interstitial deletion of the long arm of the second chromosome 11 homologue, in addition to the aberrations found in stemline (**Figure 1b**). Metaphase FISH analysis, using the 13q14.3/13q34 probe set, identified the chromosome 13 hybridization signals on the derivative chromosome 3 with a diminished hybridization signal for D13S319 resulting from partial loss of D13S319 at the 13q14.3 breakpoint. Also, Fluorescence *in situ* hybridization (FISH) analyses revealed loss of *ATM* in 93% (186/200) of interphase nuclei and loss of D13S319 in 65% (130/200) of interphase nuclei (**Figures 1c, d**). The FISH signal pattern was normal for trisomy 12 (*DDIT3*) and *TP53* (**Figures 1e, f**). Cytogenetic studies from the follow-up in July 2021 revealed a normal 46,XY karyotype, and FISH analysis was negative for both *ATM* loss and 13q deletion. However, the follow-up study conducted in July 2024 showed re-emergence of cytogenetic abnormalities. The stemline was identified as 46,XY,der(3)t(3;13)(p2?3;q14.3), der(4)t(?3;4)(p23;p16), add(11)(p13), del(13)(q14)[11], and the sideline as 46,sl,del(11)(q2?2.2q23.3)[4]. Five cells exhibited a normal karyotype. FISH analysis revealed 28.0% loss of D13S319 at 13q14.3 (28/100 cells) and 79.0% loss of *ATM* at 11q22.3 (79/100 cells). FISH was negative for trisomy 12 and *TP53* deletion.

### Optical genome mapping analysis

**Figure 2:**
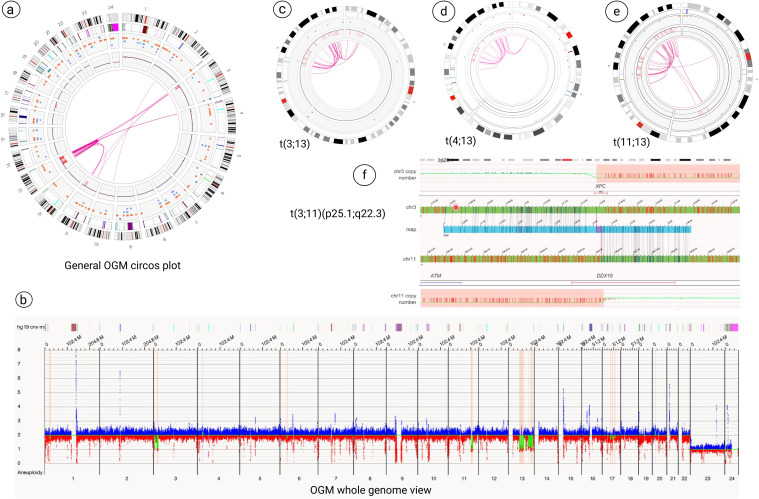
Optical genome mapping (OGM) analysis. **(a, b)** General OGM circos plot and whole genome view analysis revealed multiple structural changes, including microdeletions at 3p25 (loss of *XPC*, isoform 1), 11q22.3 (loss of *ATM* and partial loss of DDX10 genes), clusters of microdeletions from 13q14.2 to 13q33.3 and microdeletions on the long arm of chromosome 17. **(c-e)** OGM technology revealed chromothripsis of chromosome 13 along with chromoplexy between chromosomes 3, 4, 11 and 13. **(f)** The breakpoints of t(3;11)(p25.1;q22.3) showed the two cancers genes of *XPC* and *DDX10* that are involved in this rearrangement.

**Figure 3:**
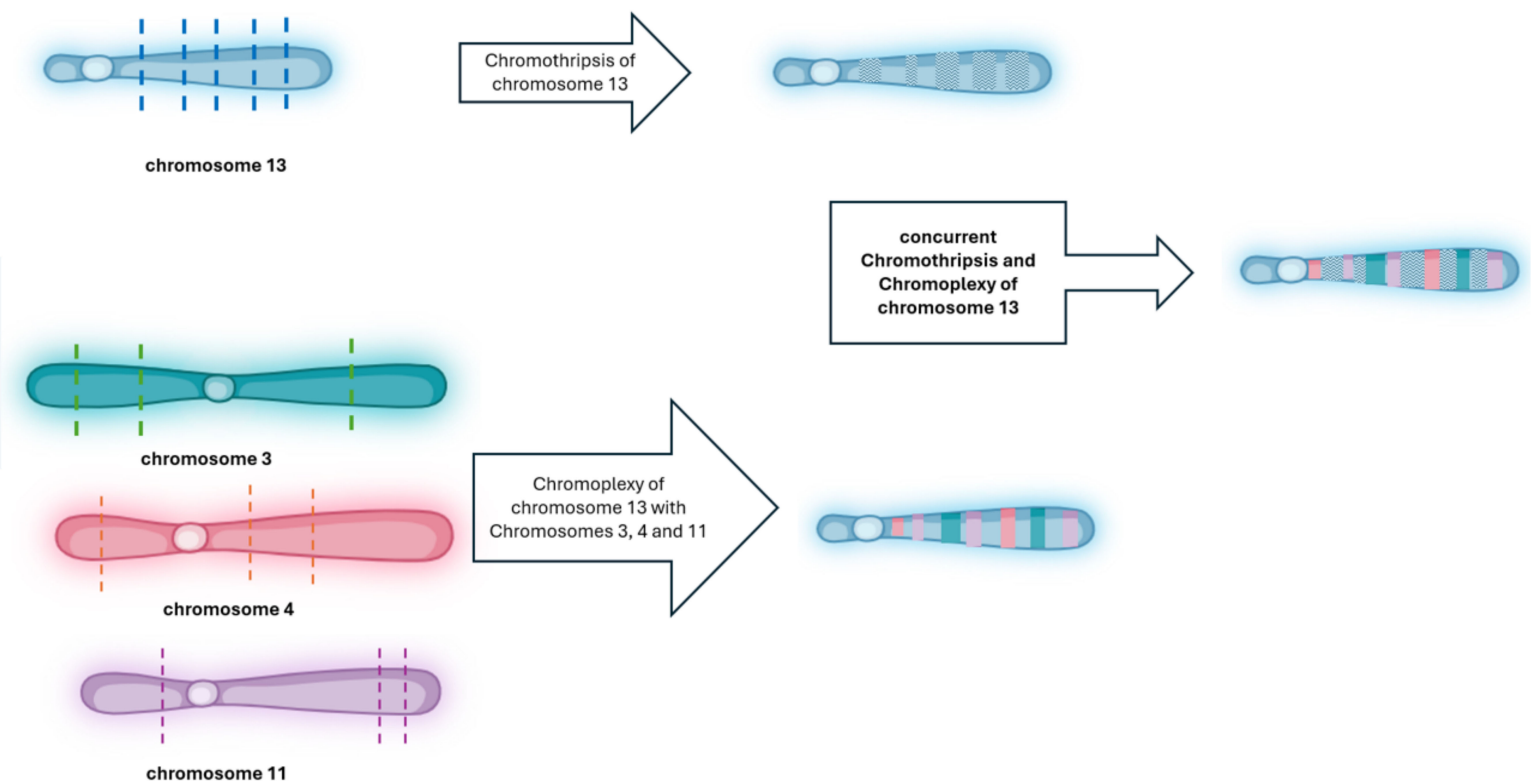
Schematic illustration: chromothripsis and chromoplexy involving chromosome 13 in CLL. This diagram illustrates the sequential and combined structural rearrangements observed in chromosome 13. In the top panel, chromothripsis of chromosome 13 is depicted, initiated by multiple DNA double-strand breaks (indicated by blue dashed lines), followed by random reassembly of the fragmented segments, resulting in a disordered and patterned appearance (shown with hatched regions). In the bottom panel, chromoplexy is illustrated as a complex event involving chromosome 13 and chromosomes 3 (green dashed lines), 4 (orange dashed lines), and 11 (purple dashed lines). These interchromosomal double-strand breaks lead to the formation of a highly rearranged derivative chromosome composed of fragments from all four chromosomes, shown as multi-colored segments. The central panel shows the outcome of concurrent chromothripsis and chromoplexy involving chromosome 13, where chromothriptic fragments are additionally involved in chained translocations with other chromosomes, resulting in a highly complex and mosaic chromosome architecture. Dashed lines indicate predicted breakpoints, and colored blocks represent rearranged segments derived from multiple chromosomes. This figure was created by Biorender (app.biorender.com).

OGM analysis revealed multiple structural changes, including:

- Microdeletions at 3p26.1, 3p25.1 (loss of *XPC*, isoform 1), 3p24.3, 11q22.3 (loss of *ATM* and partial loss of *DDX10* genes), 11q22.3-q23.1, chromothripsis with clusters of microdeletions from 13q14.2 to 13q33.3 (Figure in **Supplementary Material**) and 17q12-q21.2, 17q21.31-q21.33, 17q22-q23.1 (**Figures 2a, b**).
- Three t(3;13): ogm[GRCh38] t(3;13)(p25.1;q32.1)(16,261,873~16,263,221;95,252,504~95,280,390)[VAF0.49], ogm[GRCh38] t(3;13)(p24.3;q21.1)(18,634,486~18,647,506;55,830,924~55,855,288)[VAF0.49], ogm[GRCh38] t(3;13)(p24.3;q32.1)(20,114,179~20,133,873;95,296,771~95,302,001)[VAF0.39] (**Figure 2c**).
- Two t(4;13): ogm[GRCh38] t(4;13)(p15.31;q32.1) (20,869,721~20,907,265;96,617,837~96,630,317)[VAF0.42], ogm[GRCh38] t(4;13)(p15.31;q33.2) (20,838,804~20,849,946;105,703,642~105,710,730)[VAF0.51] (**Figure 2d**).
- Three t(11;13): ogm[GRCh38] t(11;13)(q22.3;q33.1)(103,078,067~103,090,407;102,591,041~102,602,147)[VAF0.43], ogm[GRCh38] t(11;13)(q22.3;q31.1)(103,434,662~103,442,300;80,863,864~80,899,634)[VAF0.46], ogm[GRCh38] t(11;13)(q23.1;q31.1)(111,537,352~111,546,712;80,110,741~80,127,397)[VAF0.42] (**Figure 2e**).
- Chromothripsis of chromosome 13 (Figure in **Supplementary Material**) along with chromoplexy between chromosomes 3, 4, 11 and 13 (**Figures 2c-e**, **Figure 3**).
- Additionally, the breakpoints of t(3;11)(p25.1;q22.3) translocation, ogm[GRCh38]t(3;11)(p25.1;q22.3)(14,123,898~14,176,062;108,739,990~108,747,294)involved two cancers genes of *XPC* and *DDX10* (**Figure 2f**).

## Discussions

CLL has a variable clinical course, with outcomes influenced by each patient’s unique molecular and cytogenetic profile. This clinical and genetic heterogeneity is key to guiding treatment and prognosis (17). For instance, short telomeres are linked to unmutated *IGHV* (U-IGHV) status, high-risk genomic aberrations, and a poor prognosis (17, 18). Over time, CLL evolves as therapy-resistant subclones with additional genetic anomalies emerge, leading to relapse and a more aggressive disease course (17). Acquired chromosomal abnormalities occur in about 80% of CLL patients, making cytogenetic markers essential for prognostic classification (19). FISH analysis of the four-probe set from Dohner’s hierarchical model is considered the gold standard for cytogenetic evaluation in CLL (10, 20), with five prognostic categories: the poorest prognosis is associated with a 17p deletion (*TP53* gene), followed by an 11q22.3 deletion (*ATM* gene), trisomy 12 and normal FISH results. The most favorable prognosis is seen in patients with a 13q deletion as the sole abnormality (20–22).

Complex karyotype (CK), defined as the presence of three or more chromosomal abnormalities, is observed in 15%–20% of newly diagnosed CLL cases, but its prevalence increases to 30%–40% in relapsed or refractory cases (17). Additionally, common molecular alterations in CLL patients include mutations in genes of *NOTCH1* (10-15%), *ATM* (10-15%), *SF3B1* (10%), *TP53* (5-10%), and *MYD88* (3-8%) (19). The molecular results of our patient showed unmutated *IGHV*, wild-type *TP53*, *ATM* loss and mutation in *XPO1* (c.1711G>A; p.E571K) gene.

The emergence of optical genome mapping (OGM) has significantly enhanced the sensitivity and specificity of the cytogenomics studies as compared to traditional methods such as karyotyping, FISH and chromosomal microarray analysis (CMA). OGM enables comprehensive whole-genome analysis, detecting the structural variants ranging from 500 bp to 500 kb that may be missed by other methods. It is particularly effective in identifying complex chromosomal rearrangements and balanced abnormalities, which karyotype, and CMA often fail to detect. While whole genome sequencing methods have been widely used to characterize complex rearrangements (5, 13, 23, 24), challenges such as high costs, the need for extensive data storage, and the requirement for advanced bioinformatics analysis pipelines limit their incorporation into routine practice (5, 9). Conversely, several studies have highlighted the use of OGM in detecting additional abnormalities, including complex chromosomal rearrangements such as chromothripsis and chromoplexy, in hematological malignancies (5, 9).

Chromothripsis has been observed in 1-5% of CLL cases and is typically linked to a poor prognosis (5, 11). Chromothripsis was associated with significantly shorter time to first treatment (TTFT), yet multivariate analysis revealed that *TP53* mutations or deletions, rather than chromothripsis itself, were the dominant predictors of adverse outcomes (9). This suggests that while chromothripsis contributes to genomic instability and possibly clonal selection, functional *TP53* may buffer its pathogenic consequences. Conversely, *TP53* disruption likely permits the expansion of genomically unstable subclones, driving early treatment failure and poor prognosis (9). Notably, chromothripsis has been shown to persist despite treatment (5, 9), suggesting that it represents a stable and early genomic event that is not easily eliminated by therapeutic intervention. This observation aligns with our patient’s findings, in which the same stemline and sideline karyotypes, along with FISH-detected losses of ATM and 13q, persisted following therapy. This persistence suggests that chromothriptic rearrangements may be resistant to treatment-induced selective pressures and could represent a foundational genomic alteration within the malignant clone. The mechanisms underlying this resistance and stability remain unclear, highlighting the need for further investigation into the role of chromothripsis in clonal maintenance and disease progression.

Various patterns of chromothripsis-related rearrangements involving different chromosomes have been reported. In some instances, the highly complex profiles may be associated with chromoplexy, characterized by multiple chained translocations, similar to the pattern observed in our patient (9). Chromoplexy is a highly complex genomic profile characterized by multiple chained and interconnected translocations across several chromosomes. Initially discovered in prostate cancer, it has since been observed in other tumors as well (6, 9). Although they have distinct characteristics, the exact distinction between chromothripsis and chromoplexy remains unclear.

Detection of combined complex rearrangements of both chromothripsis and chromoplexy is extremely rare in CLL and has been reported in a few cases (12, 13). In this study we found chromothripsis of chromosome 13 along with chromoplexy between chromosomes 3, 4, 11 and 13 in our CLL patient, which is similar to a few previously described CLL cases (12, 13). While chromothripsis and chromoplexy have been reported in CLL cases, particularly with the application of high-resolution techniques like whole-genome sequencing (WGS) and Optical Genome Mapping (OGM), their long-term clinical impact and relationship to treatment response remain insufficiently defined (9–13, 17). According to the literature, these complex rearrangements occur more frequently in *IGHV*-unmutated tumors and are often, but not exclusively, associated with *TP53* aberrations, which complicates the interpretation of their independent prognostic relevance. Notably, some cases, including ours, show these events in the absence of *TP53* disruption, raising the possibility of *TP53*-independent mechanisms driving complexity and progression. For example, while cases with both *IGHV*-unmutated status and *TP53* mutations were associated with more aggressive disease (13), one CLL patient with co-occurring chromothripsis and chromoplexy but wild-type *TP53* remained clinically stable for over two years (9). As observed during follow-up, our patient with co-occurring chromothripsis, chromoplexy and wild-type *TP53* remained clinically stable from July 2021 through October 2024. These observed clinical and genomic heterogeneity, suggests that other molecular or epigenetic modifiers may influence disease course. Therefore, larger, longitudinal studies are needed to systematically evaluate the prognostic and therapeutic implications of chromothripsis and chromoplexy in CLL, particularly in the context of intact *TP53*. Such studies may help determine whether these rearrangements act as independent markers of clinical outcome or contribute to therapy resistance and clonal evolution.

Furthermore, it is crucial to recognize the limitations of Optical Genome Mapping (OGM) in cytogenomic studies, particularly in cancer. One key limitation of OGM is its limited ability to fully resolve clonal evolution, particularly in heterogeneous samples. As CLL progresses or responds to treatment, the clonal composition of the disease can shift, with emerging subclones potentially present at low frequencies. OGM has a relatively high limit of detection for structural variants, making it less sensitive to minor subclonal populations that may be critical for understanding disease dynamics, relapse, or resistance. As a result, while OGM provides a high-resolution snapshot of the dominant genomic landscape at a given time, it may not effectively capture the full spectrum of evolving subclonal architectures.

Additionally, the detection of structural abnormalities using Optical Genome Mapping (OGM) can be challenging in the context of minimal residual disease (MRD), where the proportion of abnormal cells is often very low. OGM typically requires a minimum variant allele frequency or cell fraction to reliably identify structural variants. In post-treatment MRD scenarios, leukemic cells may fall below this detection threshold, potentially resulting in false-negative findings or underrepresentation of clinically relevant genomic alterations. These limitations underscore the need for complementary methods with higher sensitivity—such as PCR-based assays or next-generation sequencing (NGS)—to effectively monitor low-level disease. Therefore, OGM results in samples with low-abundance subclones must be interpreted with caution and may require additional validation using other techniques to ensure diagnostic accuracy (9, 10).

Despite these limitations, Optical Genome Mapping (OGM) has emerged as a powerful and highly informative tool for cytogenomic analysis in chronic lymphocytic leukemia (CLL) patients. Its ability to provide an unbiased, genome-wide assessment of structural variants (SVs) and copy number variations (CNVs) at high resolution has significantly enhanced our understanding of the complex genetic landscape of CLL. By detecting cryptic chromosomal rearrangements, chromothripsis, chromoplexy, and other complex genomic events that may not be captured by conventional cytogenetic methods, OGM allows for a more refined classification of genetic subtypes and risk stratification. This, in turn, enables a more accurate prognostic assessment and may help in guiding personalized therapeutic approaches. Additionally, OGM’s ability to detect structural alterations in key driver genes and pathways associated with disease progression and treatment resistance provides valuable insights into clonal evolution and disease trajectory. Although its clinical utility is still evolving, its potential to redefine prognostic markers and improve patient management in CLL is undeniable.

In conclusion, chromosome 13q deletion is a recurrent and frequently observed chromosomal abnormality in CLL patients. In this case analyzed by Optical Genome Mapping (OGM), the findings were unexpected. OGM not only revealed chromothripsis affecting chromosome 13 but also uncovered chromoplexy involving chromosome 13 and chromosomes 3, 4, and 11. This significantly elevated the level of complexity from what initially appeared to be a simple terminal deletion of the long arm of chromosome 13 and unbalanced t(3;13) translocation to a highly intricate pattern of genomic rearrangements. The utility of OGM in deciphering genomic alterations is clearly demonstrated in this case. OGM enables precise visualization of complex structural variations, providing deeper insights into genomic architecture, prognostic implications, and potential novel cancer-related genes. This case highlights OGM’s value in refining clinical risk assessment and enhancing our understanding of the broader genomic landscape in CLL.

## Data Availability

The authors acknowledge that the data presented in this study must be deposited and made publicly available in an acceptable repository, prior to publication. Raw data have been deposited in Zenodo with DOI 10.5281/zenodo.15742069. Frontiers cannot accept a article that does not adhere to our open data policies.

## Supplementary material

Supplementary Figure 1 Copy number variation (CNV) analysis of chromosome 13 by optical genome mapping showing the chromothripsis of chromosome 13. Regions of copy number loss appear as dips below the baseline (in red). Several distinct regions of loss are observed, such as 13q14.3–q21.1, 13q21.1–q21.31, 13q21.31–q21.32, 13q31.2–q31.3, and 13q33.2–q33.3, highlighted with red bars and arrows. Chromosomal position is represented on the X-axis in megabases (Mb), and relative copy number is plotted on the Y-axis. These findings point to genomic imbalances observed as chromothripsis and complex rearrangements.

## References

[1] Zepeda-Mendoza CJ Morton CC . The iceberg under water: unexplored complexity of chromoanagenesis in congenital disorders. Am J Hum Genet. (2019) 104:565–77. doi: 10.1016/j.ajhg.2019.02.024, PMID: 30951674 PMC6451730

[2] Holland AJ Cleveland DW . Chromoanagenesis and cancer: mechanisms and consequences of localized, complex chromosomal rearrangements. Nat Med. (2012) 18:1630–8. doi: 10.1038/nm.2988, PMID: 23135524 PMC3616639

[3] Forment JV Kaidi A Jackson SP . Chromothripsis and cancer: causes and consequences of chromosome shattering. Nat Rev Cancer. (2012) 12:663–70. doi: 10.1038/nrc3352, PMID: 22972457

[4] Wang JC Fisker T Sahoo T . Constitutional chromothripsis involving chromosome 19 in a child with subtle dysmorphic features. Am J Med Genet A. (2015) 167A:910–3. doi: 10.1002/ajmg.a.36962, PMID: 25736334

[5] Stephens PJ Greenman CD Fu B Yang F Bignell GR Mudie LJ . Massive genomic rearrangement acquired in a single catastrophic event during cancer development. Cell. (2011) 144:27–40. doi: 10.1016/j.cell.2010.11.055, PMID: 21215367 PMC3065307

[6] Baca SC Prandi D Lawrence MS Mosquera JM Romanel A Drier Y . Punctuated evolution of prostate cancer genomes. Cell. (2013) 153:666–77. doi: 10.1016/j.cell.2013.03.021, PMID: 23622249 PMC3690918

[7] Liu P Erez A Nagamani SC Dhar SU Kolodziejska KE Dharmadhikari AV . Chromosome catastrophes involve replication mechanisms generating complex genomic rearrangements. Cell. (2011) 146:889–903. doi: 10.1016/j.cell.2011.07.042, PMID: 21925314 PMC3242451

[8] Salaverria I Martin-Garcia D Lopez C Clot G Garcia-Aragones M Navarro A . Detection of chromothripsis-like patterns with a custom array platform for chronic lymphocytic leukemia. Genes Chromosomes Cancer. (2015) 54:668–80. doi: 10.1002/gcc.22277, PMID: 26305789 PMC4832286

[9] Ramos-Campoy S Puiggros A Kamaso J Bea S Bougeon S Larrayoz MJ . TP53 abnormalities are underlying the poor outcome associated with chromothripsis in chronic lymphocytic leukemia patients with complex karyotype. Cancers (Basel). (2022) 14(15):3715. doi: 10.3390/cancers14153715, PMID: 35954380 PMC9367500

[10] Puiggros A Ramos-Campoy S Kamaso J de la Rosa M Salido M Melero C . Optical genome mapping: A promising new tool to assess genomic complexity in chronic lymphocytic leukemia (CLL). Cancers (Basel). (2022) 14. doi: 10.3390/cancers14143376, PMID: 35884436 PMC9317182

[11] Zavacka K Plevova K . Chromothripsis in chronic lymphocytic leukemia: A driving force of genome instability. Front Oncol. (2021) 11:771664. doi: 10.3389/fonc.2021.771664, PMID: 34900721 PMC8661134

[12] Valkama A Vorimo S Kumpula TA Rasanen H Savolainen ER Pylkas K . Optical genome mapping as an alternative to FISH-based cytogenetic assessment in chronic lymphocytic leukemia. Cancers (Basel). (2023) 15. doi: 10.3390/cancers15041294, PMID: 36831635 PMC9953986

[13] Puente XS Bea S Valdes-Mas R Villamor N Gutierrez-Abril J Martin-Subero JI . Non-coding recurrent mutations in chronic lymphocytic leukaemia. Nature. (2015) 526:519–24. doi: 10.1038/nature14666, PMID: 26200345

[14] Jeffet J Margalit S Michaeli Y Ebenstein Y . Single-molecule optical genome mapping in nanochannels: multidisciplinarity at the nanoscale. Essays Biochem. (2021) 65:51–66. doi: 10.1042/EBC20200021, PMID: 33739394 PMC8056043

[15] Banu S Mk K George JK Siby E Bhagat R Ms S . Enhanced resolution of optical genome mapping utilizing telomere-to-telomere reference in genetic disorders. Eur J Hum Genet. (2025) 33(7):956–959. doi: 10.1038/s41431-024-01763-z, PMID: 39653745 PMC12229659

[16] Tsai MM Kao HJ Chen HH Yu CH Chien YH Hwu WL . Optical genome mapping with whole genome sequencing identifies complex chromosomal structural variations in acute leukemia. Front Genet. (2025) 16:1496847. doi: 10.3389/fgene.2025.1496847, PMID: 40242470 PMC12000080

[17] Serafin A Ruocco V Cellini A Angotzi F Bonaldi L Trentin L . Management strategies for patients with chronic lymphocytic leukaemia harbouring complex karyotype. Br J Haematol. (2025). doi: 10.1111/bjh.19986, PMID: 39761654 PMC11886947

[18] Rampazzo E Bonaldi L Trentin L Visco C Keppel S Giunco S . Telomere length and telomerase levels delineate subgroups of B-cell chronic lymphocytic leukemia with different biological characteristics and clinical outcomes. Haematologica. (2012) 97:56–63. doi: 10.3324/haematol.2011.049874, PMID: 21933855 PMC3248931

[19] Wainman LM Khan WA Kaur P . Chronic lymphocytic leukemia: current knowledge and future advances in cytogenomic testing. In: Sergi CM , editor. Advancements in Cancer Research. Brisbane (AU): Exon Publications (2023), Chapter 6., PMID: 37756426

[20] Dohner H Stilgenbauer S Benner A Leupolt E Krober A Bullinger L . Genomic aberrations and survival in chronic lymphocytic leukemia. N Engl J Med. (2000) 343:1910–6. doi: 10.1056/NEJM200012283432602, PMID: 11136261

[21] Van Dyke DL Shanafelt TD Call TG Zent CS Smoley SA Rabe KG . A comprehensive evaluation of the prognostic significance of 13q deletions in patients with B-chronic lymphocytic leukaemia. Br J Haematol. (2010) 148:544–50. doi: 10.1111/j.1365-2141.2009.07982.x, PMID: 19895615 PMC2866061

[22] Shanafelt TD Witzig TE Fink SR Jenkins RB Paternoster SF Smoley SA . Prospective evaluation of clonal evolution during long-term follow-up of patients with untreated early-stage chronic lymphocytic leukemia. J Clin Oncol. (2006) 24:4634–41. doi: 10.1200/JCO.2006.06.9492, PMID: 17008705

[23] Cortes-Ciriano I Lee JJ Xi R Jain D Jung YL Yang L . Comprehensive analysis of chromothripsis in 2,658 human cancers using whole-genome sequencing. Nat Genet. (2020) 52:331–41. doi: 10.1038/s41588-019-0576-7, PMID: 32025003 PMC7058534

[24] Burns A Alsolami R Becq J Stamatopoulos B Timbs A Bruce D . Whole-genome sequencing of chronic lymphocytic leukaemia reveals distinct differences in the mutational landscape between IgHV(mut) and IgHV(unmut) subgroups. Leukemia. (2018) 32:332–42. doi: 10.1038/leu.2017.177, PMID: 28584254 PMC5808074

