# Correction: Case Report: Unraveling complex genomic alterations in a case of chronic lymphocytic leukemia using optical genome mapping

**DOI:** 10.3389/fonc.2025.1707033

**Published:** 2025-11-13

**Authors:** Leila Youssefian, Sahoo Trilochan, Jia-Chi Wang

**Affiliations:** 1 Cytogenetics Laboratory, City of Hope National Medical Center, Irwindale, CA, United States; 2 Bionano Laboratories, San Diego, CA, United States

**Keywords:** fluorescence *in situ* hybridization (FISH), chronic lymphocytic leukemia (CLL), optical genome mapping (OGM), chromothripsis and chomoplexy, complex karyotype (CK), structural variants (SV), genome instability, cancer cytogenetics

The **Conflict of interest** statement was erroneously given as “Author TS was employed by the company Bionano Genomics, Inc. The remaining authors declare that the research was conducted in the absence of any commercial or financial relationships that could be construed as a potential conflict of interest.”

The correct **Conflict of interest** statement is “TS was employed by the company Bionano Laboratories, San Diego. The remaining authors declare that the research was conducted in the absence of any commercial or financial relationships that could be construed as a potential conflict of interest.”

Affiliation “Bionano Laboratories, San Diego, CA, United States” was erroneously given as “Bionano Genomics, Inc., San Diego, CA, United States”.

The original version of this article has been updated.

