# Supplementary figures and images for: Case Report: Unraveling complex genomic alterations in a case of chronic lymphocytic leukemia using optical genome mapping
Source: Front Oncol. 2025 Sep 3;15:1639849. doi: 10.3389/fonc.2025.1639849 (PMC12440793; doi:10.3389/fonc.2025.1639849)

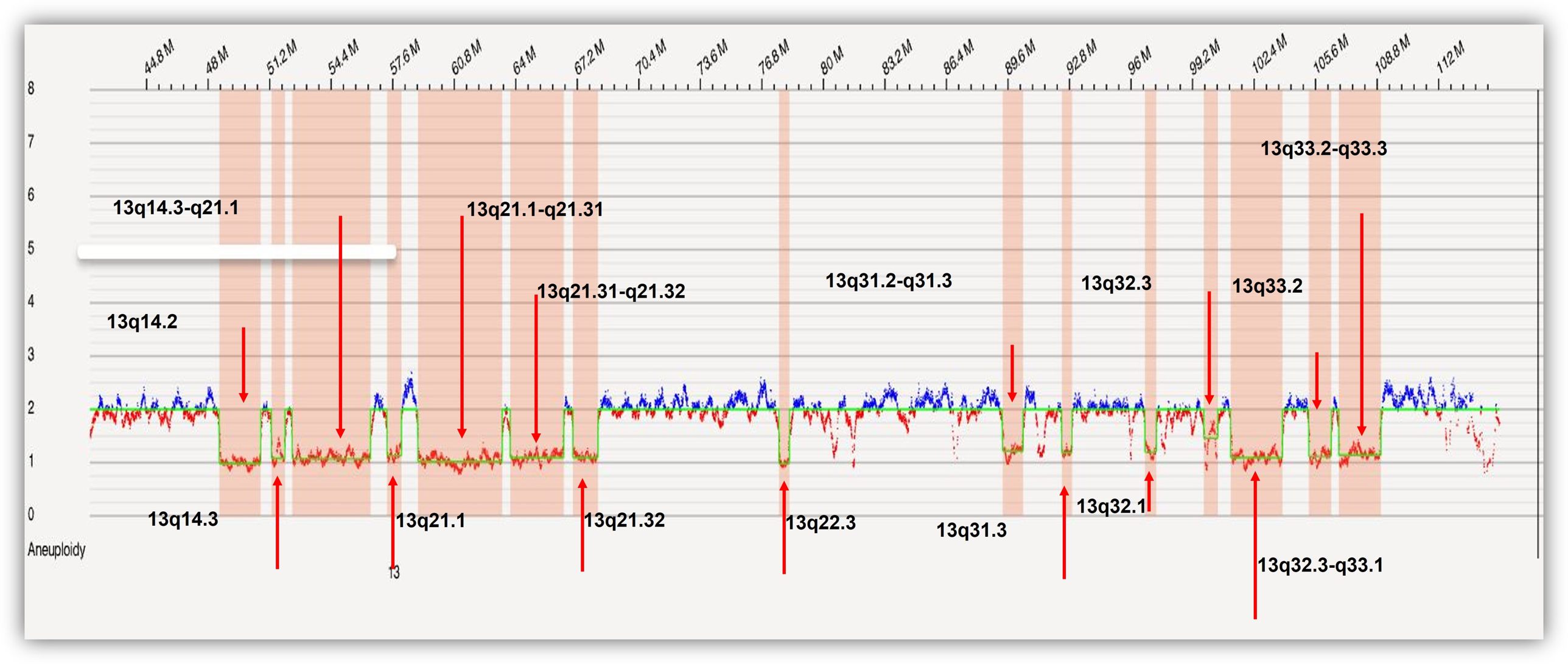

Supplement: Supplementary Figure 1 — Copy number variation (CNV) analysis of chromosome 13 by optical genome mapping showing the chromothripsis of chromosome 13. Regions of copy number loss appear as dips below the baseline (in red). Several distinct regions of loss are observed, such as 13q14.3–q21.1, 13q21.1–q21.31, 13q21.31–q21.32, 13q31.2–q31.3, and 13q33.2–q33.3, highlighted with red bars and arrows. Chromosomal position is represented on the X-axis in megabases (Mb), and relative copy number is plotted on the Y-axis. These findings point to genomic imbalances observed as chromothripsis and complex rearrangements. [file Image1.jpg]
