# Supplementary material for: Case Report: Unraveling complex genomic alterations in a case of chronic lymphocytic leukemia using optical genome mapping
Source: Front Oncol. 2025 Sep 3;15:1639849. doi: 10.3389/fonc.2025.1639849 (PMC12440793; doi:10.3389/fonc.2025.1639849)
Supplement: Supplementary file 2 [file SupplementaryFile1.pdf]

Supplementary Methods: Interpretation of OGM Output

OGM detects structural variants (SVs) by aligning ultra-long, fluorescently labeled DNA molecules to a reference genome. The output includes variant types (e.g., del, dup, inv, tra), genomic coordinates, size, allele frequency (as low as ~5%), and confidence scores. SVs are reported using standard nomenclature according to ISCN 2024. Circos plots visually summarize SVs, with chromosomes arranged in a circle, colored lines/arcs indicating inter- and intra-chromosomal events, and copy number changes displayed as concentric bar graphs.
